# MPC1 and MPC2 expressions are associated with favorable clinical outcomes in prostate cancer

**DOI:** 10.1186/s12885-016-2941-6

**Published:** 2016-11-16

**Authors:** Xiaoli Li, Yasai Ji, Gaoyang Han, Xiaoran Li, Zhirui Fan, Yaqing Li, Yali Zhong, Jing Cao, Jing Zhao, Mingzhi Zhang, Jianguo Wen, Mariusz Adam Goscinski, Jahn M. Nesland, Zhenhe Suo

**Affiliations:** 1 Department of Oncology, The First Affiliated Hospital of Zhengzhou University, Zhengzhou, Henan Province China; 2 Department of Thoracic Surgery, The First Affiliated Hospital of Xinxiang Medical University, Weihui City, Henan Province China; 3 Department of Pathology, The Norwegian Radium Hospital, Oslo University Hospital, Institute of Clinical Medicine, University of Oslo, Montebello, Oslo, Norway; 4 Department of Pathology, The Third Affiliated Hospital of Zhengzhou University, Zhengzhou, Henan Province China; 5 The Institute of Clinical Medicine, The First Affiliated Hospital of Zhengzhou University, Zhengzhou, Henan Province China; 6 Departments of Surgery, The Norwegian Radium Hospital, Oslo University Hospital, Institute for Clinical Medicine, Faculty of Medicine, University of Oslo, Montebello, Oslo, Norway

**Keywords:** MPC1, MPC2, Mitochondrial, Pyruvate, Prostate cancer

## Abstract

**Background:**

Cancer cells exhibit an altered metabolism, which is characterized by a preference for aerobic glycolysis more than mitochondrial oxidation of pyruvate. Mitochondrial pyruvate carrier 1 (MPC1) and mitochondrial pyruvate carrier 2 (MPC2) play a bottleneck role by transporting pyruvate into mitochondrial through the mitochondrial inner membrane. Therefore, their protein expression in cancers may be of clinical consequences. There are studies showing low levels of MPC1 expression in colon, kidney and lung cancers, and the expression of MPC1 correlates with poor prognosis. However, the expression status of MPC1 and MPC2 in prostate cancer (PCA) is unclear.

**Methods:**

In this study, expression of MPC1 and MPC2 in LNCaP and DU145 prostate cancer cell lines was examined by immunocytochemistry (ICC) and Western blotting. Compared to the LNCaP cells, lower levels of MPC1 and MPC2 expression in the DU145 cell line was identified. We then extended our study to 88 patients with prostate cancer who underwent transurethral electro-vaporization of prostate or radical prostatectomy at the First Affiliated Hospital of Zhengzhou University, Henan, China. Patient-derived paraffin embedded PCA specimens were collected for immunohistochemistry (IHC). Correlations with clinicopathologic factors were evaluated by Chi-square or Fisher´s exact probability tests. Overall survival (OS) rates were determined using the Kaplan-Meier estimator. The Cox proportional hazard regression model was used in univariate analysis and multivariate analysis to identify factors significantly correlated with prognosis.

**Results:**

Linear regression analysis revealed that MPC1 expression level was positively correlated with MPC2 expression (*r* = 0.375, *P* = 0.006) in the prostate cancers. MPC1 expression was negatively associated with UICC stage (*P* = 0.031). While UICC stage (*P* < 0.001) and lymph node metastasis (*P* = 0.002) were negatively associated with MPC2 expression. Positive MPC1 or MPC2 expression in cancer tissues was significantly associated with higher OS (*P* < 0.05). The multivariate analysis showed that both MPC1 and MPC2 expressions in PCA were independent prognostic factors for higher OS (For MPC1: RR = 0.654, 95% CI: 0.621-0690, *P* < 0.001; For MPC2: RR = 0.696, 95% CI: 0.660-0.734, *P* < 0.001).

**Conclusions:**

Our study indicates that MPC1 and MPC2 expressions are of prognostic values in PCAs and that positive expression of MPC1 or MPC2 is a predictor of favorable outcome.

## Background

Prostate cancer (PCA) is one of the most common cancers and the sixth leading cause of cancer death among men throughout the world [1]. Over the last decade, the morbidity of PCA was steadily increased in China, due to the changing in dietary pattern and Westernized lifestyle [2]. Nowadays, serum level of prostate specific antigen (PSA), digital rectal examination (DRE) and diagnostic imaging techniques such as ultrasound and MRI are used as methods for PCA diagnosis. As a highly heterogeneous disease, PCA may vary from slow growing indolent tumor to rapidly progressing highly aggressive carcinoma, which is associated with significant morbidity and mortality [3]. It is realized now that it is important to examine the conceivable biomarkers of PCA patients to make individualized treatment possible.

Metabolism in normal condition relies on two different pathways, glycolysis and oxidative phosphorylation (OXPHOS) to generate ATP and produce energy [4]. Glycolysis is a process that converts glucose into lactate, which generates 2 molecules of ATP per molecule of glucose. In normoxia condition, cellular glucose is converted into pyruvate, which is carried into mitochondrial and oxidized, a process of OXPHOS, from which 36 ATP molecules are generated. Mitochondrion plays a significant role in OXPHOS. In cancer cells, aerobic glycolysis holds the main pathway to produce energy, called Warburg effect [5]. This way is quicker and suitable for cancer tissues proliferation [6]. Although it yields less ATP than OXPHOS, this is more suitable for the growth of cancer cells, since higher energy production may worsen the body situation [7].

Pyruvate is a hub metabolite for glucose, lipid and amino acid. The cellular fate of pyruvate determines whether glycolysis is followed by OXPHOS, or by lactic fermentation. It has been known that the existence of mitochondrial pyruvate carrier allows the pyruvate entering into the mitochondrial matrix, and the functions of the MPC molecules are recently verified simultaneously by two groups [8, 9]. These studies have shown that MPC1 and MPC2 are two paralogous subunits composing the heteromeric complex of MPC in mammals, and the MPC complex is located in the inner mitochondrial membrane. Moreover, it has shown in some studies that the expression levels of MPC1 and MPC2 in cancers are decreased, and low expression is correlated with poor survival in multiple cancers, including colon, kidney and lung [10], illustrating the regulation of MPC complex is pivotal for tumor cell growth. Thus assessment of the expression of the MPC may be of significance in the understanding of cancer metabolic alterations.

In this study, we verified variable MPC1 and MPC2 expressions in two different prostate cancer cell lines (LNCaP and DU145) and found that the aggressive DU-145 cell line expressed lower levels of MPC1 and MPC2. Then we extended our study in analyzing the expression status of MPC1 and MPC2 in a series of 88 PCA samples, aiming to explore their clinicopathological and survival correlations.

## Methods

### Cell lines

LNCaP and DU145 cell lines were purchased directly from American Type Culture Collection (ATCC), USA in 2006. The cells were expanded for 4 passages, and all the cells were preserved in nitrogen before use in this study. All the cells were authenticated in October 2016 by Sangon Biotech Co., Ltd. (Shanghai, China) using Microreader^TM^21 ID System to analyze 9 short tandem repeat (STR) loci, showing that all these cells matched their original STR profiles. All the cells were routinely tested and confirmed to be mycoplasma free. Cells were cultivated in PRMI 1640 medium (GibcoTM, 11835-063) supplemented with 10% fetal bovine serum (FBS) (GibcoTM, 10500-064), 100units/ml penicillin and 100 mg/ml streptomycin (Life Technologies, 15140122) at 37 °C in a humidified 5% CO_2_ incubator.

### Cell block preparation

For each cell line, the cells in 80% confluent were harvested by mechanical scraping, and cells were washed twice with ice-cold phosphate-buffered saline (PBS) and collected by centrifuged at 2000 rpm for 10 minutes. Three drops of plasma and two drops of thrombin were added to the sedimentation and the contents were carefully mixed by rotating tube for one minute until the coagulation was formed. 4% buffered formaldehyde was added to the coagulation for cell fixation in 30 minutes. The coagulated mass was then wrapped in a linen paper, put in a labeled cassette and placed in 4% buffered formaldehyde. The material was paraffin-embedded to make cytoblock before being cut into 4 mm paraffin sections for immunocytochemistry (ICC).

### Western blotting

All the cells were harvested by cell scraper when cells grew 80% confluent and the cells in suspension were centrifuged at 1000 rpm for 5 minutes. After washed with ice-cold PBS twice, the cells were dissolved with lysis buffer containing RIPA buffer (Thermo scientific, 89900) and 1% protease inhibitor cocktail (Thermo scientific, 1862209) by pipetting gently up and down, put on ice before spun down at 13000 rpm for 10 minutes at 4 °C to release total protein in the supernatant. Total protein concentration was measured by the Quick Start^TM^ Bradford (Bio-Rad, 500-0205). Equal amount of proteins from each sample in sodium dodecyl sulfate (SDS) loading buffer was boiled for 10 minutes at 100 °C, and the protein samples were subjected to 10% SDS-PAGE electrophoresis and then electro-transferred to high-quality polyvinylidene difluoride (PVDF) membrane in a Trans-Blot apparatus (Bio-rad, Hercules, CA). The membrane was blocked with 5% fat-free milk for 2 hour at room temperature and incubated overnight at 4 °C with rabbit anti-human MPC1 antibody (1:500, NOVUS, NBP1-91706) and MPC2 antibody (1:1000, Abcam, ab10391). After washing with TBST (TBS with 0.1% Tween), the blot was incubated with corresponding secondary antibodies conjugated with horseradish peroxidase-conjugated (HRP) for 2 hours at room temperature. Finally, the blot was visualized using an enhanced chemiluminescence detection kit (ECL, Amersham) and analyzed by Image Lab 2.0 Software (Bio-Rad Laboratories Inc, USA). The protein band was normalized to α-Tublin.

### Patients

Paraffin embedded samples from 88 PCA patients were enrolled in this study. All the patients were admitted to the First Affiliated Hospital of Zhengzhou University from December 2005 to December 2011. Inclusion criteria: (1) not received surgical resection or radio-/chemo-/hormonal treatment before tissue collection; (2) with full information of clinical/TNM staging; (3) with confirmed diagnosis with prostate cancer by postoperative pathological examination. The detailed clinicopathological features are summarized in Table 1. The ages rank from 55 to 92 years old (average age = 71 years). A further TNM staging following the American Joint Committee on Cancer (AJCC) standard identified 67 stage II patients, 21 stage III and IV patients. Lymph node metastasis was discovered in 14 patients. A further differentiation score based on Gleason system [11] showed 27 low (<7), 41 moderate (=7) and 20 (>7) high grade tumors. The distance metastasis was identified in 25 cases. Median prostatic specific antigen (PSA) level: 77.56 ng/ml (0.2–100.00). Patients were followed up from the confirmed date of diagnosis until death or 1 Jan. 2015. Two pathologists at the Department of Pathology of the First Affiliated Hospital of Zhengzhou University reviewed and diagnosed all the specimens.

**Table 1:** Clinical and pathologic characteristics for 88 patients with malignant prostate cancer

Variable:	Median (range) or NO. of patient
Age:	71 years (55–92)
Preoperative PSA:	77.56 ng/ml (0.2–100.00)
Follow-up:	51 months (3–111)
Gleason score:	
<7	27
7	41
>7	20
TNM staging:	
pT2	67
pT3-pT4	21

### Immunocytochemistry (ICC) and Immunohistochemistry (IHC)

ICC and IHC detection of MPC1 and MPC2 were performed with the use of the Dako Envision FLEX+ system (K8012, Dako, Glostrup, Denmark). Paraffin sections were deparaffinized. Microwaving antigen retrieval was performed in citrate buffer (pH 6.0) for 15 min then returned to room temperature and washed in PBS. Blocking was operated by peroxidase blocking (Dako) for 5 minutes. The slides were incubated at 4 °C overnight with MPC1 antibody (1:700, NOVUS, NBP1-91706) and MPC2 antibody (1:300, Abcam, ab10391), following with second antibody linker incubation for 15 minutes before HRP was added and incubated for 30 minutes at room temperature. Slides were then stained with 3, 39-diaminobenzidine tetrahydrochloride (DAB) for 10 minutes and counterstained with hematoxylin, dehydrated and mounted.

### IHC scoring system

MPC1 and MPC2 immunodetections were evaluated by two pathologists, who were blinded to the outcomes of patients. The scores were grouped according to intensity and extent of staining. The extent of positivity was scored as follows: 0, no positive cells; 1, <10% positive cells; 2, 10–50% positive cells; and 3, >50% positive cells. The intensity was scored as follows: 0, no positive cells; 1, weak staining; 2, moderate staining; and 3, strong staining. The immunohistochemical staining score was multiplying extent by intensity (0, 1, 2, 3, 4, 6 or 9). For statistical analyses, a score of 0 was designated negative, the score of 1 and 2 as weakly positive, and the score of 3-9 as positive.

### Statistical analyses

SPSS 17.0 software (SPSS Inc, Chicago, IL, United States) was used for data analyses. Associations between categorical variables were assessed by Chi-square tests (Pearson as appropriate) or Fisher`s exact probabilities. The relationship between MPC1 and MPC2 expressions was evaluated by linear regression analysis. Survival analysis was estimated using the Kaplan-Meier method, and groups were compared with log-rank tests. For all the analyses, associations were considered to be significant if the *P* value was smaller than 0.05. Cox regression method was used to analyze the factors of prognosis.

## Results

### Expression of MPC1 and MPC2 in prostate cancer cell lines

**Fig. 1:**
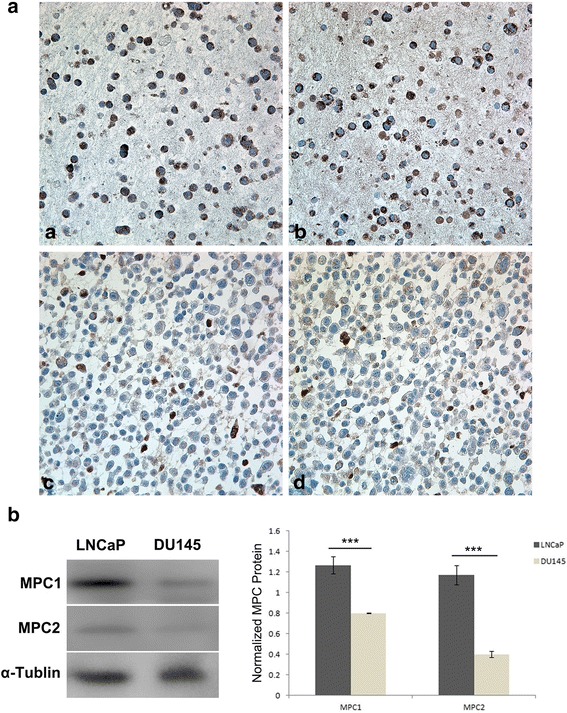
ICC and Western blotting of MPC1 and MPC2 expression in prostate cancer cell lines. **a**: Strong MPC1 (a) and MPC2 (b) immunoreactivities in the LNCaP cell line; Weak MPC1 (c) and MPC2 (d) protein expression in the DU145 cell line. All the photos were taken at 400X. **b**: Similar levels of MPC1 and MPC2 proteins revealed by Western blotting in these cell lines are shown as revealed with ICC shown in A, i.e.: low expression of both MPC1 and MPC2 in DU145 cell line, compared to the protein expression in LNCaP cell line. α–tubulin was used as loading control. Right penal shows quantified denstitometry of the Western blottings. Data are presented as mean ± SEM (n = 3 separate test). Statistical significance: ***P < 0.001

ICC identified variable MPC1 and MPC2 protein expressions in the prostate cancer cell lines LNCaP and DU145 (Fig. 1A, a, b, c, d). Comparatively, it was discovered that DU145 cell line showed the lowest expression of both MPC1 and MPC2 (Fig. 1A c, d), and LNCaP cell line was strongly positive (Fig. 1A a, b) for these two proteins. Similar levels of the MPC1 and MPC2 protein expressions were confirmed by Western blotting in these cell lines as well, with immunoreactive bands of 10 kDa and 12 kDa for MPC1 and MPC2, respectively (Fig. 1b).

### MPC expression in human PCA tissues

**Fig. 2:**
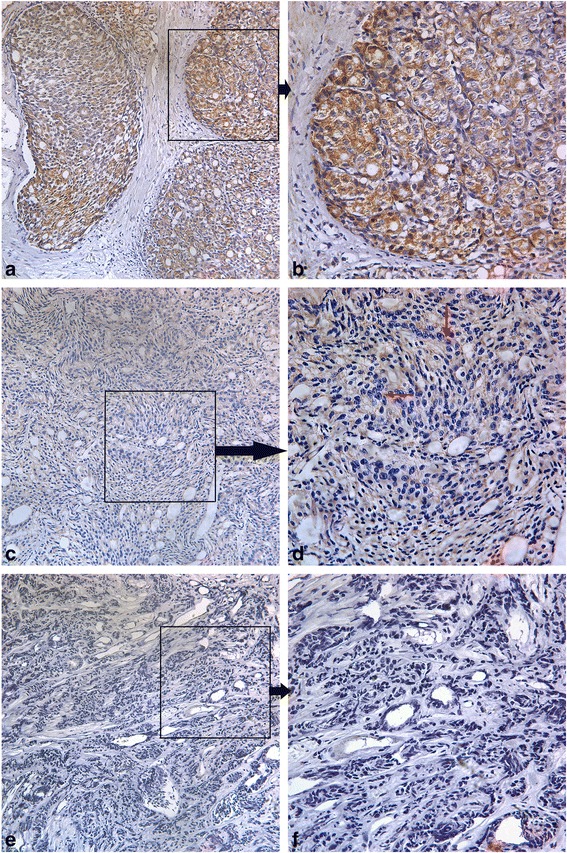
Immunohistochemical staining of MPC1 in prostate cancer samples. The typical diffuse cytoplasmic staining of the protein can be found in prostate cancer. **a, b**: MPC1 strong positivity was observed in the cytoplasm of prostate cancer cells; **c, d**: MPC1 weak positivity was observed in the cytoplasm of prostate cancer cells; **e, f**: MPC1 negativity was observed in the cytoplasm of prostate cancer cells. The dark arrows show that where the images in the right panel come from. The red arrows point to the tumor cells with weakly positive MPC1 protein expression. Magnification in the left panel: 200X; Magnification in the right panel: 400X

**Fig. 3:**
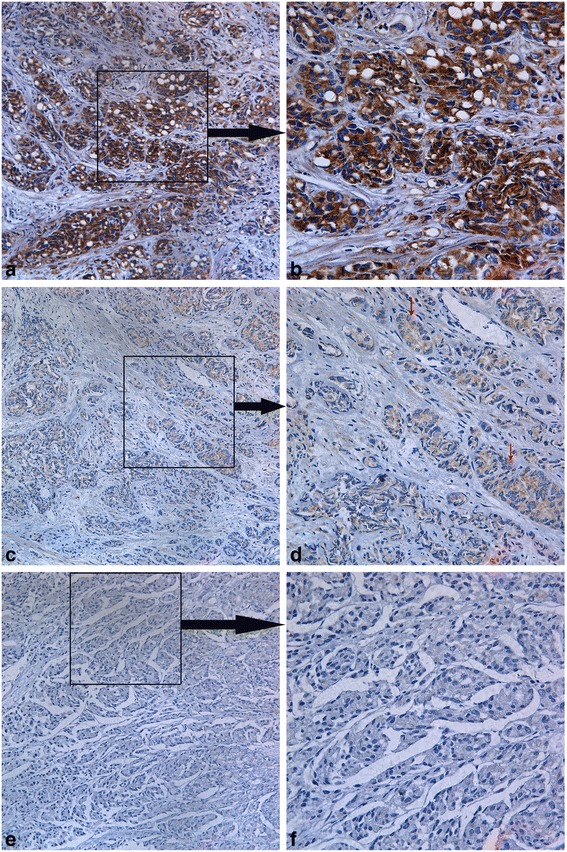
Immunohistochemical staining of MPC2 in prostate cancer samples. The typical diffuse cytoplasmic staining of the protein can be found in prostate cancer. **a, b**: MPC2 strong positivity was observed in the cytoplasm of prostate cancer cells; **c, d**: MPC2 weak positivity was observed in the cytoplasm of prostate cancer cells; **e, f**: MPC2 negativity was observed in the cytoplasm of prostate cancer cell. The dark arrows show that where the images in the right panel come from. The red arrows point to the tumor cells with weakly positive MPC1 protein expression. Magnification in the left panel: 200X, Magnification in the right panel: 400X

Both MPC1 and MPC2 immunohistochemical reactivities were confined to cytoplasm of cells. Typical diffused cytoplasmic staining of the MPC1 protein is shown in Fig. 2 and Fig. 3 shows typical cytoplasmic MPC2 expression in a PCA. It was discovered that a large number of tumors were negative for either MPC1 or MPC2, or for both proteins. Out of the 88 tumors, 29(33.33%) were positive for MPC1 protein expression, while 23(26.14%) were positive for MPC2 protein expression. Linear regression analysis further revealed that the MPC1 expression was positively correlated with the MPC2 expression in the PCA tumor tissues (*r* = 0.348, *P* =0.017; Table 2).

**Table 2:** Linear regression analysis of MPC1 and MPC2 expression in PCA

MPC2	MPC1				*P* ^1^ value	r^2^
	Positive	Weak positive	Negative	Total		
Positive	13	9	1	23		
Weak positive	15	23	11	49		
Negative	1	13	2	16		
Total	29	45	14	88	0.006	0.375

^1^Pearson Chi-Square test

^2^Contingency coefficient

### Clinicopathological correlation

The associations between MPC1 and MPC2 protein expression and the clinicopathological features were analyzed. As summarized in Table 3, MPC1 expression was significantly negatively associated with UICC stage (*P* < 0.05). MPC1 protein positive expression was noted only in 2/21 (9.52%) pT3- pT4 stage samples. No significant association was found between the MPC1 protein expression and other clinical parameters such as age, Gleason score, lymph node metastasis, PSA and distant metastasis. Table 4 shows that MPC2 expression is significantly negatively associated with UICC stage and lymph node metastasis (*P* < 0.05). 11/21 (52.38%) of the pT3- pT4 PCA samples were weakly positive and negative for the MPC2 protein expression. The MPC2 protein expression was negatively associated with lymph node metastasis, and 13 out of the 14 (92.86%) tumors with lymph node metastases were either weakly positive or negative for the protein.

**Table 3:** Relationship between MPC1 expression and clinicopathological features of prostate cancer

Clinicopathologic	n	MPC1 expression			
Variable		Positive	Weak positive	Negative	*P* value^1^
	88	29(32.95%)	45(51.14%)	14(15.91%)	
Age(year)					0.221
≤71	45	11(24.44%)	26(57.78%)	8(17.78%)	
>71	43	18(41.86%)	19(44.19%)	6(13.95%)	
Gleason score					0.682
<7	27	10(37.04%)	14(51.85%)	3(11.11%)	
7–10	61	19(31.15%)	31(50.82%)	11(18.03%)	
PSA (ng/ml)					0.715^a^
≤10	10	4(40.00%)	4(40.00%)	2(20.00%)	
> 10 and ≤ 20	10	2(20.00%)	6(60.00%)	2(20.00%)	
> 20	68	23(33.8%)	35(51.47%)	10(14.71%)	
UICC stage					0.031
pT2	67	27(40.30%)	31(46.27%)	9(13.43%)	
pT3-pT4	21	2(9.52%)	14(66.67%)	5(23.81%)	
lymph node metastasis					0.288
Negative	74	24(32.43%)	40(54.05%)	10(13.51%)	
Positive	14	5(35.71%)	5(35.71%)	4(28.57%)	
distant metastasis					0.386
Negative	63	19(30.16%)	32(50.79%)	12(19.05%)	
Positive	25	10(40.00%)	13(52.00%)	2(8.00%)	

^1^Pearson Chi-Square test; ^a^ Fisher’s exact probabilities test

**Table 4:** Relationship between MPC2 expression and clinicopathological features of prostate cancer

Clinicopathologic	n	MPC2 expression			
Variable		Positive	Weak positive	Negative	*P* value^1^
	88	23(26.14%)	49(55.68%)	16(18.18)	
Age(yr)					0.652
≤71	45	10(22.22%)	27(60.00%)	8(17.78%)	
>71	43	13(30.23%)	22(51.16%)	8(18.60%)	
Gleason score					0.367
<7	27	9(33.33%)	12(44.44%)	6(22.22%)	
7–10	61	14(22.95%)	37(60.66%)	10(16.39%)	
PSA (ng/ml)					0.763^a^
≤10	10	4(40.00%)	5(50.00%)	1(10.00%)	
> 10 and ≤ 20	10	1(10.00%)	5(50.00%)	4(40.00%)	
> 20	68	18(26.48%)	39(57.35%)	11(16.18%)	
UICC stage					0.000
pT2	67	13(19.40%)	47(70.15%)	7(10.45%)	
pT3-pT4	21	10(47.62%)	2(9.52%)	9(42.86%)	
lymph node metastasis					0.002
Negative	74	22(29.73%)	43(58.11%)	9(12.16%)	
Positive	14	1(7.14%)	6(42.86%)	7(50.00%)	
distant metastasis					0.939
Negative	63	17(26.98%)	35(55.56%)	11(17.46%)	
Positive	25	6(24.00%)	14(56.00%)	5(20.00%)	

^1^Pearson Chi-Square test; ^a^ Fisher’s exact probabilities test

### Decreased MPC1 and MPC2 expressions in PCA are associated with unfavorable survivals

**Fig. 4:**
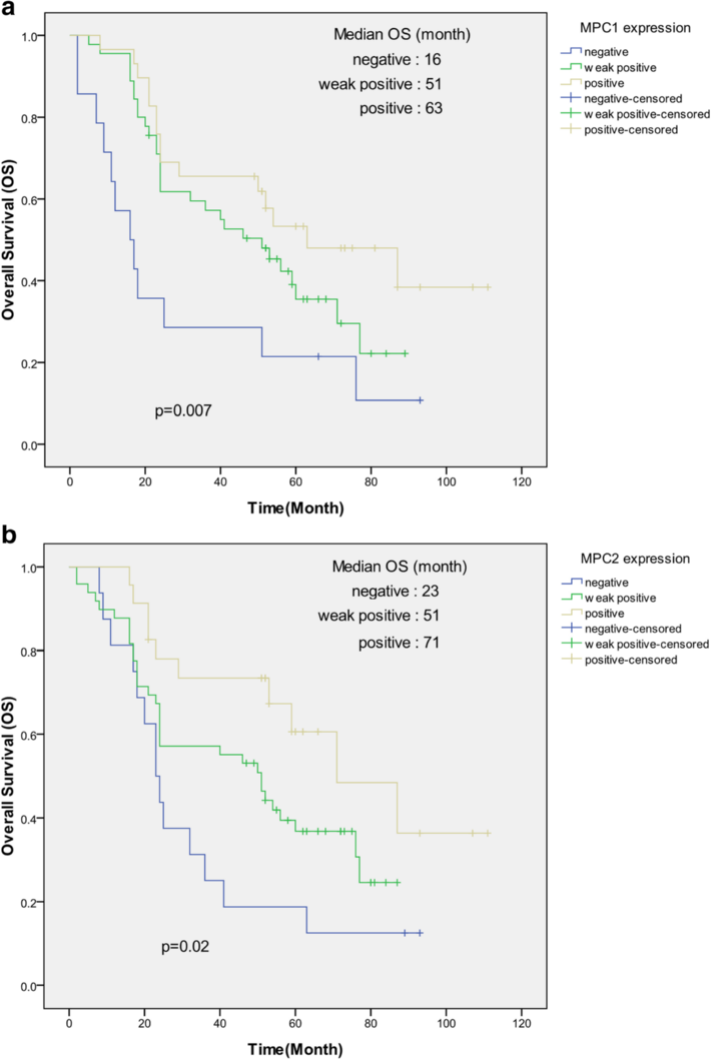
Correlation between MPC1 and MPC2 expression and prognosis of prostate cancer patients. **a**: Kaplan-Meier survival curves show the positive expression of MPC1 is significantly associated with a better overall survival in PCA. **b**: The Kaplan-Meier curves for overall survival rate stratified by MPC2 show that negative MPC2 protein expression is significantly associated with shorter OS survival in PCA patients

The overall survival (OS) rate of the 88 patients with PCA was 36.4%, with 56 deaths observed during the follow-up period. The median duration of follow-up was 51 months (ranging from 3 to 111 months). Kaplan-Meier survival curves and the log-rank test demonstrated that patients with positive expression of MPC1 in the tumor had significantly better OS than the patients with negative MPC1 expression in the tumor (*P* =0.007; Fig. 4a). The survival rate of patients with positive MPC1 protein expression was significantly higher than that of patients with weak positive and negative MPC1 protein expression (48.3% v.s. 30.5%, respectively). Similarly, patients with positive expression of MPC2 in the tumor had significantly better OS than did patients with negative MPC2 expression in the tumor (*P* =0.02; Fig. 4b) according to Kaplan-Meier survival curves and the log-rank test. The survival rate of patients with positive MPC2 protein expression was also significantly higher than that of patients with lower MPC protein expression (56.5% v.s. 29.2%, respectively).

### MPC1 and MPC2 expression are independent risk factors for overall survival

Univariate analysis and multivariate analysis were performed using Cox proportional hazards regression method on the above clinicopathological parameters with MPC1 and MPC2 expression in tumor (Table 5). UICC stage (RR = 1.198, 95% CI: 1.095-1.311, *P* < 0.001),PSA (RR = 1.091,95% CI: 1.040-1.143,*P* < 0.001) and Gleason score (RR = 1.635, 95% CI: 1.514-1.765, *P* < 0.001) are independent risk factors for overall survival in prostate cancer patients. Moreover, MPC1 and MPC2 expressions are also independent prognostic factors for overall survival in PCA (For MPC1: RR = 0.654, 95% CI: 0.621-0.690, *P* < 0.001; For MPC2: RR = 0.696, 95% CI: 0.660-0.734, *P* < 0.001), while other variables including age, PSA, lymph node metastasis and distant metastasis did not contribute to overall survival independently (*P* > 0.05).

**Table 5:** Univariate and multivariate analysis for overall survival using Cox relative risk

Variable	Univariate analysis		Multivariate analysis	
	RR (95% CI)	*P* value^1^	RR (95% CI)	*P* value^1^
MPC1 expression in tumor	0.561	0.000	0.654	0.000
	(0.536-0.588)		(0.621-0.690)	
MPC2 expression in tumor	0.558	0.000	0.696	0.000
	(0.533-0.585)		(0.660-0.734)	
Age	0.977	0.000	0.998	0.371
(≤71 year vs >71 year)	(0.973-0.981)		(0.994-1.002)	
UICC stage	1.34	0.000	1.198	0.000
(pT2 vs pT3-pT4)	(1.259-1.427)		(1.095-1.311)	
Gleason score	1.769	0.000	1.635	0.000
(< 7, 7-10 )	(1.645-1.902)		(1.514-1.765)	
PSA(ng/ml)	1.344	0.000	1.091	0.000
(≤10, > 10 and ≤ 20,>	(1.286-1.405)		(1.040-1.143)	
lymph node metastasis	1.361	0.000	0.985	0.742
(positive vs negative)	(1.253-1.478)		(0.897-1.080)	
distant metastasis	1.094	0.008	1.049	0.293
(positive vs negative)	(1.023-1.170)		(0.960-1.147)	

*RR* relative risk; 95%CI: 95% confidence interval; ^1^Cox regression

## Discussion

Normal adult cells maximize ATP production by metabolizing glucose through the OXPHOS pathway in the mitochondria. However, the prostate is an exception. The prostate epithelium is unique in its ability to produce, accumulate and release large amounts of citrate into prostatic fluid [12]. But the level of citrate found in PCA is significantly reduced. The different concentrations of citrate between normal prostate and PCA indicate that PCA cells may have ability to use citrate for metabolic energy production [13], or the main pathway for citrate synthesis is impeded.

Forty years ago, a study postulated the existence of a mitochondrial pyruvate carrier that allows pyruvate entry into the mitochondrial matrix [14]. And it was revealed in 2012 that two paralogous subunits, MPC1 and MPC2, were expressed in mammals and formed a multimeric MPC complex that controls pyruvate transportation, which were originally known as BRP44L and BRP44 [8, 9, 15]. Studies have shown that when overexpressing either MPC1 or MPC2 by itself in colorectal cancer cells, the protein fails to accumulate to a high level, suggesting that these two proteins might need to form a complex to be stable [10]. Another study found that the native complex showed an apparent molecular weight of 150 kDa in blue native gels, while the theoretical molecular weight of a dimeric MPC complex would be around 30 kDa, indicating that multiple dimmers assemble to form the mature carrier [16]. In humans, mutations in MPC1 have been identified and associated with defects in mitochondrial pyruvate metabolism, lactic acidosis, hyperpyruvatemia, severe illness and failure to thrive [8, 17]. Since its discovery, interest in the MPC complex as a drug target for cancer, neurological disorders, and metabolic diseases has been extremely high. Thus, a better understanding of MPC expression has the potential to advance our knowledge and impact drug discover for current public problems.

Several studies have examined the MPC activity of tumor by using different methodologies, and reduced MPC function in various cancers has been reported [18–20]. Metabolic studies by using radiolabelled pyruvate and hyperpolarized ^13^C-enriched substrates to monitor pyruvate metabolism have shown reduced MPC metabolism pathway in cancers [18, 19]. Low activity of MPC in cancer cells is also reported in a real time engineered biosensor monitoring study [20].

Aerobic glycolysis is a hallmark of tumor cell metabolism, and MPC has a transporter role that facilities the pyruvate through the mitochondrial inner membrane. Our present report was to assess the localization and expression status of MPC1 and MPC2, and further explore their clinicopathological correlations in a series of human PCA specimens. Firstly, we detected MPC1 and MPC2 expression in the two human prostate cancer cell lines by ICC and Western blotting, confirming the different expressions of MPC1 and MPC2 in various histological subtype derived cell lines. LNCaP cells are androgen-sensitive human prostate adenocarcinoma cells derived from the left supraclavicular lymph node metastasis, expressed the highest level of MPC1 and MPC2. While the cell line DU145 is of Androgen Insensitive (AI) state, and this cell line has lower levels of MPC1 and MPC2 proteins. The LNCaP cells always show low metastatic potential, as compared to the DU-145 cells [21]. The expression of MPC1 or MPC2 in these cell lines indicates a potential clinical role of MPC in PCA.

Although mitochondria have subsequently been shown to be vital for cancer growth [22, 23], we have shown herein that the MPC expression in PCA patient tissue is reduced. The inclusion of the MPC adds additional complexity to targeting cancer metabolism for therapy but has the potential to explain why treatments may be more effective in some studies than in others [24–27]. An important finding in our current study is that positive expression of MPC1 as well as MPC2 is associated with good survival in PCA patients. These findings are consistent with previous studies that the positive expression of MPC has a better survival in colon cancer [10].

Our data have also demonstrated that there is a positive relationship between the expression levels of MPC1 and MPC2 in PCA (*r* = 0.375, *P* = 0.006). This is consistent with the conclusion that loss of either MPC1 or MPC2 protein results in the destabilization and degradation of the other and thus loss of the MPC complex [28, 29]. It is known that knockdown of MPC1 in prostate cancer cells increases glycolysis and cell invasion [30]. Increased glycolysis has long been demonstrated to promote cancer progression through many ways [31, 32]. Recently, repression of MPC1 expression is found not only to increase glycolysis through blocking glucose-derived pyruvate entering into mitochondria, but also to increase the supply of compensatory TCA cycle intermediates from glutamine, amino acids and fatty acids [19, 33]. The TCA cycle and glycolysis provide a synthetic precursor for lipids, proteins and nucleic acids. MPC1 down-regulation mimics a glucose-starved circumstance, which mobilizes or activates usage of different fuel sources to maintain the high levels of precursor pools for cell proliferation, thus promoting cancer progression.

Next, we analyzed the correlation between MPC1 and MPC2 expressions and clinicopathological parameters including clinical outcomes. Our study shows that tumor tissue MPC2 expression is inversely correlated with the following PCA clinicopathologic characteristics like UICC stage and lymph node metastasis, while MPC1 expression is inversely correlated with the UICC stage. Therefore, we propose that the loss of MPC expression may contribute to poorly differentiated PCAs. Another research team found that MPC1 expression is much lower in the primary tumors than in the normal adjacent benign prostate tissues, and is further down-regulated in metastatic prostate tumors, indicating that MPC1 down-regulation may predict a more aggressive prostate cancer. More importantly, patients having low levels of MPC1 expression showed poor prognosis in prostate cancer [30]. Consistently, we analyzed the prognostic role of MPC1 and MPC2 on OS of patients with PCA and found a significant association between MPC expression and OS of patients, and patients with positive MPC1 or MPC2 protein expression had a longer survival time.

Furthermore, multivariate analysis suggested that the expressions of MPC1 and MPC2 can be independent predictive factors for PCA. These results are consistent with the previous studies, suggesting that MPC may serve as a new therapeutic target for prostate cancer.

## Conclusions

In conclusion, we have found low expression of MPC1 and MPC2 in agressive prostate cancer cell line, and also discovered that negative expression of MPC complex is significantly associated with unfavorable clinicopathological features in PCA samples and shorter survival in PCA patients. However, the disadvantages in our studies are the limited number of samples and relatively short follow-up period, which should be explained with care. Moreover, the clinical correlation of MPC1 and MPC2 in PCA disclosed herein clearly merits further elucidations. The MPC1 and MPC2 variants and their potential molecular and biological functions in human prostate carcinoma are currently under investigation in our laboratory.

## Abbreviations

95%CI: 95% confidence interval
AI: Androgen Insensitive
AJCC: American Joint Committee on Cancer
DAB: 3, 39-diaminobenzidine tetrahydrochloride
DRE: digital rectal examination
HRP: horseradish peroxidase-conjugated
ICC: immunocytochemistry
IHC: immunohistochemistry
MPC1: mitochondrial pyruvate carrier 1
MPC2: mitochondrial pyruvate carrier 2
OS: Overall Survival
OXPHOS: glycolysis and oxidative phosphorylation
PBS: phosphate-buffered saline
PCA: prostate cancer
PSA: prostate specific antigen
PVDF: polyvinylidene difluoride
RR: relative risk
SDS: sodium dodecyl sulfate
